# Bone health in transgender assigned female at birth people: effects of gender-affirming hormone therapy and gonadectomy

**DOI:** 10.3389/fendo.2024.1416121

**Published:** 2024-09-26

**Authors:** Elena Sanna, Alessandra Lami, Giulia Giacomelli, Stefania Alvisi, Alexandro Paccapelo, Renato Seracchioli, Maria Cristina Meriggiola

**Affiliations:** ^1^ Division of Gynecology and Human Reproduction Physiopatology, Istituito di Ricovero e Cura a Carattere Scientifico (Scientific Institute for Research, Hospitalization and Healthcare) (IRCCS) Azienda Ospedaliero-Universitaria di Bologna, Bologna, Italy; ^2^ Department of Medical and Surgical Sciences (DIMEC), University of Bologna, Bologna, Italy; ^3^ Research and Innovation Unit, IRCCS Azienda Ospedaliero-Universitaria di Bologna, Bologna, Italy

**Keywords:** bone health, transgender, assigned female at birth (AFAB), testosterone, Dual-energy X-ray absorptiometry (DXA), gender affirming hormonal therapy (GAHT), bilateral oophorectomy (BO), bone mineral density (BMD)

## Abstract

**Introduction:**

Gender-affirming hormone therapy (GAHT) and gender-affirming surgery (GAS) may be desired by transgender and gender-diverse (TGD) individuals who want to affirm their gender identity. Testosterone is the basis of GAHT for transgender individuals assigned female at birth (AFAB), whereas GAS can involve hysterectomy, bilateral salpingectomy, bilateral oophorectomy (BO), thorax masculinization, and phalloplasty. Our study aimed to evaluate the effects of GAHT on the bone health of TGD AFAB individuals who have undergone or not undergone BO.

**Methods:**

This was a single-center, longitudinal study with retrospectively collected data. TGD AFAB GAHT-naïve individuals were enrolled and underwent dual-energy X-ray absorptiometry scans and laboratory tests (hormonal and bone metabolism parameters) at baseline and after 5 and 10 years of GAHT.

**Results:**

Two hundred and forty-three TGD AFAB people were included in this study. Seventy-five subjects had completed data for 5 years and 19 subjects for 10 years of GAHT. At baseline, low bone density (Z-score < –2.0) was found in 2.5% (6/243) of subjects for lumbar spine (LS), whereas total hip (TH) and femoral neck (FN) Z-scores and laboratory tests were within the normal female range. After stratifying by physical activity, the physically active group showed significantly higher LS BMD and Z-scores (p ≤ 0.05). Five years after the start of GAHT, a significant reduction in LS (p ≤ 0.05), TH (p ≤ 0.001), and FN (p ≤ 0.01) Z-scores was detected. A significant reduction in the Z-scores of all three bone sites was observed only in the subgroup that had undergone BO. After 5 years of GAHT, estradiol levels were significantly decreased compared to those in baseline (p ≤ 0.001). Significantly higher estradiol levels were detected in the 5-year no-BO subgroup compared to those in the 5-year BO subgroup (p ≤ 0.001). A significant reduction in LS and TH Z-scores were observed after 10 years of GAHT. At this time, estradiol levels were significantly decreased compared to those in baseline (p ≤ 0.01).

**Conclusion:**

Bone density in TGD AFAB individuals is comparable to that in their peers prior to GAHT and BO, but those subjects who underwent BO had a reduced Z-score at LS, FN, and TH after 5 years and at LS after 10 years of GAHT.

## 1 Introduction

Gender incongruence is a condition characterized by the incongruence between an individual’s experienced gender and the gender assigned at birth. To affirm their gender identity, transgender and gender-diverse (TGD) people may require social transition, psychological support, pubertal suppression, gender-affirming hormone therapy (GAHT), and/or some kind of gender-affirming surgery (GAS) (1). There is no universal model, and transgender individuals can undergo all, some, or none of these treatments to achieve the affirmation of their experienced gender (2).

In TGD individuals assigned female at birth (AFAB), GAHT involves the administration of testosterone (T) through injectable or transdermal routes to induce virilization (voice tone deepening, increased body hair, and changes in body composition). GAS may include hysterectomy, bilateral salpingectomy, bilateral-oophorectomy (BO), genital reconstructive surgery and thorax masculinization.

Given the crucial role of sex hormones in bone physiology, some attention has been directed toward understanding the impact of GAHT on bone metabolism over the years whereas data regarding the possible effects of bilateral oophorectomy (BO) is scant (3–5). In adult life, estrogens (Es) contribute to the maintenance of bone mineral density (BMD) and trabecular bone mass through their pro-apoptotic effects on osteoclasts and their activating effects on osteoblasts (6). Indirect beneficial effects of T on bone health are attributed to its peripheral conversion into estradiol (E2) by the aromatase enzyme. In this regard, lower E2 levels due to the use of aromatase inhibitors in gonadectomized TGD AFAB individuals undergoing T treatment are shown to result in a reduction in BMD (7). Another indirect beneficial effect of T is related to muscle mass: the increase in lean muscle mass and strength creates a greater mechanical load on bones, potentially stimulating bone formation. Therefore, GAHT may influence bone health in TGD adults undergoing gender-affirming treatment. Various cross-sectional and longitudinal studies have suggested that T can maintain BMD in TGD AFAB individuals and, indeed, may even offer beneficial effects for those who are in peri-postmenopause (4, 8). However, a reduction of BMD has been reported in gonadectomized TGD AFAB people due to irregular GAHT uptake or lower E2 levels (9–11).The aim of our study was to report the modifications in bone health parameters in TGD AFAB individuals undergoing GAHT and BO over 10-year follow-up.

## 2 Materials and methods

### 2.1 Study design and population

This was a single-center, longitudinal study with data collected retrospectively conducted from 2008 to 2023 at the Gynecology and Human Reproduction Pathophysiology Unit of the IRCCS Sant’Orsola-Malpighi, Azienda Ospedaliera Universitaria di Bologna. This study was approved by the Medical Ethics Committee of the Area Vasta Emilia Centro (CE-AVEC), and all subjects gave a written informed consent.

Inclusion criteria included the following: diagnosis of gender dysphoria according to Diagnostic and Statistical Manual of Mental Disorders-4th edition (DSM-4) or DSM-5 criteria according to the year of inclusion; age between 14 and 60; and GAHT naïve at the time of enrollment. Exclusion criteria were as follows: sex chromosome anomalies; having previously undergone hysterectomy or BO; current assumption of GAHT; use of medication that affects the hypothalamic pituitary ovarian axis, including those used to induce amenorrhea; and preexisting bone diseases or rheumatological conditions.

During the initial visit, the following parameters were recorded: age, body mass index (BMI), medical history, medication intake, smoking habit, alcohol consumption habit, physical activity, dietary habits, and occurrence of fractures.

TGD people were divided into sedentary and physically active, according to WHO recommendations for adults: physically active were considered people who undertake at least 150–300 min of moderate-intensity aerobic physical activity or at least 75–150 min of vigorous-intensity aerobic physical activity throughout the week (12) Enrolled participants underwent anthropometric measurements (height, weight, and BMI), bone fracture history, dual-energy X-ray absorptiometry (DXA) scans, and laboratory tests at baseline, after 5 (range, 4–6) and 10 (range, 9–11) years from the start of GAHT. Clinical data were prospectively collected for all subjects during in-clinic visits.

GAS considered in this study as relevant to bone metabolism consisted of BO. Transgender AFAB individuals who had undergone BO received GAHT with no change of the dose after surgery.

The primary endpoint of our study was to evaluate changes in BMD in patients undergoing GAHT with T after 5 and 10 years compared to those in baseline and according to BO. The secondary endpoint was to assess hormonal and bone marker changes over the same time frame.

### 2.2 DXA

BMD measurements were performed before the start of GAHT and were usually repeated at least every 5 years. At baseline and during follow-up (after 5 and 10 years), BMD and Z-scores were measured at IRCCS Azienda Ospedaliero-Universitaria Di Bologna using a GE Healthcare Lunar iDXA (version 16.10.151, Software Encore Version 16 - ME+200351, GE-Lunar Inc., Madison, WI, USA). BMD assessed in our study included lumbar [L1–L4 lumbar spine (LS)], total hip (TH), and femoral neck (FN) regions. Z-score was calculated on the basis of the reference tables corresponding to the assigned sex at birth, because all enrolled individuals had already passed puberty and initiated skeletal maturation under the influence of female hormones.

### 2.3 Biochemical assessment

At baseline and at least annually thereafter, serum levels of total T, E2, luteinizing hormone (LH), follicle-stimulating hormone (FSH), parathyroid hormone (PTH), bone alkaline phosphatase (BAP), 25-hydroxyvitamin D (vit. D), calcium, and phosphorus were measured. Venous blood samples were obtained after overnight fasting and analyzed with competitive immunoassays. During the study period, immunoassay measurement procedures changed due to the progressive update of equipment (7, 13–18).

### 2.4 Statistical analysis

Demographic and clinical characteristics were reported as means ± standard deviations, ranges, and frequencies. Mann–Whitney U test, Fisher’s exact test, and chi-squared test were used for baseline comparisons, and Wilcoxon signed-rank test was used for comparisons between time points. The Pearson’s correlation coefficient was computed to assess the linear correlation between BMI and DXA parameters. A p-value of <0.05 was considered significant for two-tailed tests. Statistical analysis was performed using IBM SPSS Statistics for Windows, Version 28.0 (Armonk, NY: IBM Corp). The p-values in **Table 1** were calculated on mutually exclusive groups of patients with only T0 (n = 168) of the overall group; with only T0 and T5 (n = 56) of the 5-year subgroup; and with T0, T5, and T10 (n = 19) of the 10-year subgroup.

**Table 1:** Baseline demographic and clinical characteristics of study participants in the overall group of TGD AFAB people and in the two subgroups: 5-year subgroup and 10-year subgroup.

Parameters	Overall group	5-year subgroup	10-year subgroup
Number of subjects	243	75	19
Age, mean ± SD (range)	26.5 ± 7.9 (18–55)	29.3 ± 8.1 (18–55)^**^	30.6 ± 6.4 (20–45)^**^
BMI, mean ± SD (range) kg/m2	23.3 ± 5.0 (18.2–42.9)	23.1 ± 3.9 (18.2–33.9)	22.2 ± 3.9 (18.2–32.0)
Life style, No. (%)			
Physically active	116/169 (68.6%)	46/54 (85.2%)	14/15 (93.3%)^**^
Competitive sport	27/169 (16.0%)	14/54 (25.9%)*	3/15 (20.0%)
Non-competitive sport	89/169 (52.7%)	32/54 (59.3%)	11/15 (73.3%)
Smokers	93/169 (55%)	26/54 (48.1%)	6/15 (40.0%)
< 15 cigarettes/day	54/169 (32.0%)	16/54 (29.6%)	4/15 (26.7%)
≥ 15 cigarettes/day	39/169 (23.1%)	10/54 (18.5%)	2/15 (13.3%)
Alcohol consumers	110/169 (65.1%)	41/54 (75.9%)	13/15 (86.7%)
1 U alc./day	102/169 (60.4%)	37/54 (68.5%)	11/15 (73.3%)^***^^$^
>1U alc./day	8/169 (4.7%)	4/54 (7.4%)	2/15 (13.3%)^***^^$^
Diet			
omnivorous	151/169 (89.4%)	48/54 (88.9%)	12/15 (80.0%)^$^
vegetarian	14/169 (8.2%)	5/54 (9.2%)	2/15 (13.3%)
vegan	4/169 (2.4%)	1/54 (1.9%)	1/15 (6.7%)^**^
Hormone assays, mean ± SD			
FSH mUI/mL	7.2 ± 11.4	5.9 ± 7.1*	4.6 ± 1.6^**^
LH mUI/mL	10.7 ± 12.7	8.6 ± 6.7*	7.4 ± 3.4^**^
E2 pg/mL	110.8 ± 82.7	115.5 ± 77.5	141.7 ± 111.8^*^
T ng/mL	0.6 ± 0.6	0.5 ± 0.7	0.4 ± 0.2^**^
Bone markers, mean ± SD			
BAP U/L	15.8 ± 7.3	14.4 ± 5.2	12.7 ± 6.7^*^
Vit. D ng/mL	19.0 ± 8.7	20.7 ± 9.9	22.7 ± 10.9
PTH pg/mL	42.1 ± 18.9	39.1 ± 16.9	32.7 ± 11.9^$**^
Calcium mg/dL	9.5 ± 0.4	9.5 ± 0.4	9.3 ± 0.3^*^
Phosphorus mg/dL	3.8 ± 0.6	3.9 ± 0.6	3.7 ± 0.5
DXA, mean ± SD			
BMD LS g/cm^2^	1.174 ± 0.144	1.169 ± 0.151	1.146 ± 0.135
Z-score LS	0.070 ± 1.105	0.145 ± 1.027	0.013 ± 0.912
BMD TH g/cm^2^	1.044 ± 0.134	1.031 ± 0.123	0.982 ± 0.093^**$^
Z-score TH	0.329 ± 0.980	0.335 ± 0.907	0.121 ± 0.755
BMD FN g/cm^2^	1.002 ± 0.143	0.975 ± 0.148	0.913 ± 0.114^**^ ^$^
Z-score FN	0.160 ± 1.003	0.146 ± 0.956	−0.195 ± 0.857

Data are expressed as mean ± standard deviation (SD).

VS the overall group: ^***^p-value ≤ 0.001, ^**^p-value ≤ 0.010, and ^*^p-value ≤ 0.050.

VS the 5-year subgroup: ^$^p-value ≤ 0.050.

## 3 Results

Five hundred and forty-three transgender AFAB people were followed at our center between January 2008 and January 2023. According to our inclusion/exclusion criteria, 243 were included in the study to evaluate basal bone parameters (overall group). Of these, 75 of the 243 individuals had completed data for 5 years of GAHT and were included in the data analyses at 5 years (5-year subgroup). Nineteen of the 243 individuals had completed data for 5 and 10 years of GAHT and were included in the data analyses at 10 years (10 -year subgroup). All subjects were treated with T, injectable testosterone undecanoate every 12–16 weeks or transdermal T 50 mg/day.

### 3.1 Overall group

Clinical and demographic characteristics of 243 transgender AFAB people enrolled are reported in **Table 1**. This group had 35 individuals under the age of 20, 146 individuals aged 20–29 years old, 47 individuals aged 30–39 years old, 10 individuals aged 40–49 years old, and 5 individuals aged 50–55 years old. Low bone density (Z-score < –2.0) at baseline was found in 2.5% (6/243) of the subjects for LS (L1-L4). All TH and FN Z-scores were within the normal range at baseline. At baseline, hormonal levels and bone metabolic parameters were within the normal range for the female gender except for vit. D levels, which were below the normal range (**Table 1**).

In analyses of the influence of lifestyle on densitometry parameters, no differences were observed after stratification by alcohol consumption and smoking habits nor according to vit. D levels (cutoff, 20 ng/mL). No subjects were chronically taking corticosteroids or proton pump inhibitors. After stratification according to physical activity, a significantly higher BMD and Z-score at LS level were observed in the group of physically active subjects than in those who were sedentary (**Table 2**). In relation to BMI, a linear correlation was found with BMD and Z-scores of all three bone sites examined (**Supplementary Figure 1** in **the Supplementary Materials**) (BMD: LS, p < 0.001, r = 0.301; TH, p < 0.001, r = 0.330; and FN, p < 0.001, r = 0.284; Z-score: LS, p < 0.05, r = 0.161; TH, p < 0.01, r = 0.195; and FN, p < 0.05, r = 0.154).

**Table 2:** Comparison of densitometric parameters in the overall group of TGD AFAB people (n = 169) divided according to physical activity (12).

	Sedentary (n = 53)	Physically active (n = 116)
BMD LS, g/cm^2^	1.141 ± 0.128	1.186 ± 0.155^*^
Z-score LS	−0.300 ± 0.995	0.167 ± 1.130^**^
BMD TH, g/cm^2^	1.025 ± 0.152	1.059 ± 0.137
Z-score TH	0.241 ± 1.216	0.410 ± 0.966
BMD FN, g/cm^2^	0.974 ± 0.139	1.019 ± 0.152
Z-score FN	−0.041 ± 1.036	0.211 ± 1.021

^**^p-value ≤ 0.010 and ^*^p-value ≤ 0.050.

Data are expressed as mean ± standard deviation (SD).

One hundred and seventy-nine subjects answered the questions on fractures. Of these subjects, 34.1% (61/179) reported one (88.5%, 54/61) or two (11.5%, 7/61) fractures, totaling 68 fractures. All reported fractures occurred following high-energy trauma. A total of 16.6% (12/68) of fractures occurred at the wrist, 4.4% (3/68) at the forearm, 1.5% (1/68) at the humerus, 1.5% (1/68) at the hip, and none at the spine. The remaining 75% (51/68) fractures involved other sites (hand, elbow, ribs, knee, leg, ankle, and foot).

### 3.2 Five-year subgroup

Seventy-five subjects completed 5 years of follow-up and were included in this subgroup. Clinical and demographic characteristics of this subgroup (5-year subgroup) are reported in **Table 1**. At baseline, in this subgroup, 3 individuals were under 20 years old, 42 individuals were between 20 and 29 years old, 24 individuals were between 30 and 39 years old, 4 individuals were 40–49 years old, and 2 individuals were between 50 and 55 years old. Of these, only one subject, aged 53, was in menopause at the time of enrollment. Baseline characteristics of this subgroup are not significantly different from those of the overall group, except for age: TGD AFAB people that had completed 5 years of GAHT were significantly older. At baseline, one person had a low bone density (Z-score < –2.0), 1.3%, (1/75) at LS, whereas TH and FN Z-scores were in the expected range for all other subjects (**Table 1**) At the 5-year follow-up, 57.3% (43/75) of individuals had undergone BO. The mean time from BO to the 5-year DXA scan was 28.3 ± 16.2 months.

#### 3.2.1 Hormonal measurements

After 5 years of GAHT, LH and FSH values were significantly increased, and E2 significantly decreased compared to that in baseline (**Table 3**). When divided according to the performed BO, an increase in LH and FSH levels and a decrease in E2 levels were confirmed in the subgroup that had undergone BO. Significantly higher E2 levels were detected in the 5-year no-BO subgroup compared to that in the 5-year BO subgroup. A slight but significant increase of FSH levels was also present in the 5-year no-BO subgroup due to two subjects being in spontaneous menopause at the time of the 5-year control. T levels were significantly increased after 5 years of GAHT compared to baseline regardless of surgery (**Table 3**).

**Table 3:** Comparison of hormone levels, bone markers, and densitometric parameters at baseline (T0) and after 5 years (T5) of follow-up in the 5-year subgroup of TGD AFAB people.

	5-year subgroup (n = 75)		5-year no-BO subgroup (n = 32)		5-year BO subgroup (n = 43)	
	T0	T5	T0	T5	T0	T5
FSH, mUI/mL	5.9 ± 7.1	38.2 ± 42.7^***^	5.1 ± 3.6	13.0 ± 19.9^*$$$^	6.5 ± 8.8	55.9 ± 45.5^***^
LH, mUI/ml	8.6 ± 6.7	21.0 ± 22.8^***^	8.7 ± 6.7	8.7 ± 10.2$$$	8.6 ± 6.7	29.7 ± 25.1^***^
E2, pg/mL	115.5 ± 77.5	42.7 ± 28.5^***^	137.8 ± 89.8	53.8 ± 30.0^***$$$^	97.9 ± 61.9	34.4 ± 24.7^***^
T, ng/mL	0.5 ± 0.7	4.3 ± 2.9^***^	0.5 ± 0.4	4.1 ± 2.4^***^	0.6 ± 0.8	4.5 ± 3.3^***^
BAP, UI/L	14.4 ± 5.2	16.2 ± 7.0^*^	14.5 ± 5.3	16.2 ± 6.9	14.3 ± 5.1	16.2 ± 7.2^*^
Vit. D, ng/mL	20.7 ± 9.9	23.6 ± 8.9	21.9 ± 9.5	22.8 ± 9.3	19.8 ± 10.2	24.1 ± 8.7
PTH, pg/mL	39.1 ± 16.9	38.8 ± 17.1	35.9 ± 13.1	39.1 ± 14.5	41.5 ± 19.1	38.6 ± 18.7
Calcium, mg/dL	9.5 ± 0.4	9.7 ± 0.4^***^	9.5 ± 0.4	9.7 ± 0.5*	9.4 ± 0.3	9.8 ± 0.4^***^
Phosphorus, mg/dL	3.9 ± 0.6	3.5 ± 0.7	3.9 ± 0.6	3.6 ± 0.8	3.8 ± 0.7	3.5 ± 0.5
BMD LS, g/cm^2^	1.169 ± 0.151	1.176 ± 0.131	1.170 ± 0.148	1.184 ± 0.139	1.168 ± 0.156	1.169 ± 0.126
Z-score LS	0.145 ± 1.027	−0.076 ± 0.926^*^	0.060 ± 1.042	0.028 ± 0.965	0.210 ± 1.023	−0.155 ± 0.899^*^
BMD TH, g/cm^2^	1.031 ± 0.123	1.030 ± 0.137	1.046 ± 0.115	1.057 ± 0.145	1.019 ± 0.129	1.010 ± 0.129
Z-score TH	0.335 ± 0.907	0.153 ± 0.926^***^	0.341 ± 0.885	0.319 ± 0.885	0.330 ± 0.932	0.026 ± 0.947^***^
BMD FN, g/cm^2^	0.975 ± 0.148	0.977 ± 0.142	1.002 ± 0.145	1.000 ± 0.136	0.956 ± 0.148	0.959 ± 0.145
Z-score FN	0.146 ± 0.956	−0.007 ± 1.016^**^	0.164 ± 0.791	0.203 ± 0.960	0.135± 1.058	−0.167± 1.038^**^

VS T0: ^***^p-value ≤ 0.001, ^**^p-value ≤ 0.01, and ^*^p-value ≤0.05.

VS T5 of the 5-year BO subgroup: ^$$$^p-value ≤ 0.001.

Data are expressed as mean ± standard deviation (SD).

#### 3.2.2 Bone metabolic parameters

BAP levels increased after 5 years of GAHT in the 5-year BO subgroup and maintained the same trend, without reaching statistical significance in the 5-year no-BO subgroup. No significant change was observed in PTH levels or vitamin D levels in either subgroup. A significant increase in the calcium level was observed after 5 years of GAHT (**Table 3**).

#### 3.2.3 Densitometric values

Five years after the start of GAHT, the BMD of all three sites examined did not vary from baseline values. A statistically significant reduction in the LS, TH, and FN Z-scores was detected (**Table 3**). When divided according to BO, a significant reduction in the Z-scores of all three bone sites was observed only in the 5-year BO subgroup. No significant reduction in the 5-year no-BO subgroup was detected. Furthermore, in the 5-year BO subgroup, 2.3% (1/43) showed low bone density of the LS (Z-score < –2.0), compared to that in the 5-year no-BO subgroup, in which no one reached LS Z-score < –2.0.

#### 3.2.4 Fractures

Of the TGD AFAB individuals that completed the 5 years of follow-up, 64 responded to the questions on fractures. During the 5 years of GAHT, 3 out of the 64 subjects reported a fracture. These fractures were all associated with high-energy trauma and occurred in the fingers of the hand, 66.6% (2/3) concerned people from the 5-year BO subgroup and 33.3% (1/3) concerned one person from the 5-year no-BO subgroup.

### 3.3 Ten-year subgroup

Nineteen transgender individuals with AFAB TGD completed both the 5-year and 10-year follow-ups. The clinical and demographic characteristics of this subgroup (10-year subgroup) are reported in **Table 1**. At baseline, in this subgroup, 9 subjects were between 20 and 29 years old, 9 were between 30 and 39 years old, 1 was between 40 and 49 years old, and no subject was over 50 years old. The mean age of this subgroup, the percentage of people who practice sports, and the percentage of people who consume alcohol were higher than those of the overall group. Furthermore, when it comes to dietary habits, there was a significantly lower percentage of omnivorous people and a higher percentage of vegan people compared to the overall group. A total of 89.5% of individuals in this subgroup (17/19) had undergone BO. The mean time from surgery to the 10-year DXA scan was 63.3 ± 26.2 months. At baseline, all transgender AFAB individuals in this subgroup had LS, TH, and FN Z-scores within the expected range for age and female gender (**Table 1**).

#### 3.3.1 Hormonal values

In year 10 of follow-up, in those subjects undergone BO, LH and FSH levels were significantly increased compared to those in baseline, and E2 levels were significantly decreased compared to those in baseline and year 5 of follow-up (**Table 4**). Mean LH, FSH, E2, and T levels in the two subjects who did not undergo BO were 5.4 ± 6.2 mIU/mL, 8.8 ± 5.2 mIU/mL, 41.7 ± 0.0 pg/mL, and 4.5 ± 0.0 ng/mL, respectively.

**Table 4:** Hormone levels, bone markers, and densitometric parameters at baseline (T0), after 5 (T5) and after 10 years (T10) of follow-up in the 10-year BO subgroup of TGD AFAB people.

	10-year BO subgroup (n = 17)		
	T0	T5	T10
FSH, mUI/mL	4.7 ± 1.7	59.2 ± 51.9^**^	67.3 ± 48.6^***^
LH, mUI/mL	7.5 ± 3.6	35.0 ± 31.3^**^	30.5 ± 24.0^**^
E2, pg/mL	122.5 ± 86.7	27.9 ± 12.8^**^	26.2 ± 8.2^**^
T, ng/mL	0.4 ± 0.2	4.1 ± 2.1^***^	3.1 ± 1.7^***^
BAP, UI/L	12.9 ± 7.1	14.5 ± 5.9^*^	14.3 ± 8.3
Vit. D, ng/mL	23.6 ± 11.1	22.0 ± 6.9	25.3 ± 7.1
PTH, pg/mL	32.9 ± 12.6	36.7 ± 11.5	38.5 ± 17.6
Calcium, mg/dL	9.4 ± 0.3	9.7 ± 0.5^*^	9.8 ± 0.4^**^
Phosphorus, mg/dL	3.8 ± 0.5	3.4 ± 0.5	3.5 ± 0.5
BMD LS, g/cm^2^	1.154 ± 0.136	1.142 ± 0.103	1.119 ± 0.101
Z-score LS	0.067 ± 0.917	−0.253 ± 0.645^*^	−0.535 ± 0.866^*$^
BMD TH, g/cm^2^	0.984 ± 0.091	0.995 ± 0.161	0.975 ± 0.101
Z-score TH	0.118 ± 0.727	−0.212 ± 0.682^*^	−0.206 ± 0.743
BMD FN, g/cm^2^	0.922 ± 0.117	0.913 ± 0.132	0.912 ± 0.097
Z-score FN	−0.171 ± 0.880	−0.424 ± 0.862	−0.447 ± 0.778

VS T0: ^***^p-value ≤ 0.001, ^**^p-value ≤ 0.010, and ^*^p-value ≤0.050.

VS T5: ^$^p-value ≤ 0.05.

SD, standard deviation; BMI, body mass index (calculated as weight in kilograms divided by height in meters squared); cig., cigarettes; alc., alcoholic; FSH, follicle stimulating hormone; LH, luteinizing hormone; E2, estradiol; T, testosterone; Vit. D, 25-hydroxyvitamin D; BAP, bone alkaline phosphatase; PTH, parathyroid hormone; DXA, dual-energy X-ray absorptiometry; BMD, bone mineral density; LS, lumbar spine; TH, total hip; FN, femur neck.

Data are expressed as mean ± standard deviation (SD).

#### 3.3.2 Bone metabolic parameters

After 10 years of GAHT, in comparison to the baseline levels, no significant changes were detected in other bone parameters, except for the calcium level that increased significantly compared to that in baseline (**Table 4**). Mean ± standard deviation BAP, vit. D, PTH, calcium, and phosphorus for the two people who did not undergo BO were 11.1 ± 0.5 UI/L, 13.8 ± 8.5 ng/mL, 30.3 ± 0.7 pg/mL, 9.3 ± 0.3 mg/dL, and 3.2 ± 0.0 mg/dL, respectively.

#### 3.3.3 Densitometric values

No significant changes in BMD were observed (**Table 4**). When considering the 10-year subgroup as a whole, a statistically significant reduction in LS and TH Z-scores was observed (p ≤ 0.05) after 10 years of GAHT. In the 10-year BO subgroup, a statistically significant reduction in Z-scores of the LS was observed 10 years after the start of GAHT (**Table 4**). A total of 10.5% (2/19) of the subjects had low bone density for age (Z-score <–2.0) at LS: these two subjects were part of the 10-year BO subgroup. At year 10 of GAHT, mean LS BMD, LS Z-score, TH BMD, TH Z-score, FN BMD, and FN Z-score for the two subjects who did not undergo BO were 1.028 ± 0.000 g/cm^2^, −0.800 ± 0.000, 1.002 ± 0.154 g/cm^2^, 0.467 ± 1.344, 0.847 ± 0.044 g/cm^2^, and −0.200 ± 0.849 g/cm^2^, respectively.

#### 3.3.4 Fractures

Of the subjects that completed the 10 years of follow-up, 18 answered the questions on fractures. Between years 5 and 10 of follow-up, no fracture was reported.

## 4 Discussion

In this longitudinal study with retrospectively collected data, we evaluated the effects on bone parameters of 5- and 10-year T treatment in TGD AFAB individuals. At baseline, BMD was within the normal range for age and biological sex in 237 out of 243 TGD AFAB people. In year 5, Z-score at LS, TH, and FN and in year 10 at LS showed a significant decrease in those who had undergone BO.

Our study showed that, before GAHT, most of the transgender AFAB population had bone parameters that were within the normal range of the cisgender AFAB population, in agreement with data previously reported and unlike the transgender AMAB population (3, 5). As expected, and in agreement with the AFAB cisgender population, lifestyle factors, such as physical activity and BMI, significantly influence BMD. At baseline, individuals who were physically active had better LS BMD and Z-scores than those who were sedentary (**Table 2**). We also found a linear correlation between BMI and BMD and Z-score at LS, TH, and FN that was also previously reported (8, 19, 20).

Furthermore, we examined the changes in densitometry parameters after 5 and 10 years of GAHT. We chose to use the Z-score relative to the sex assigned at birth because most of the individuals at the start of GAHT had already passed the age of peak bone mass and none had been treated with puberty blockers.

The Z-scores at all three studied bone sites decreased at years 5 and 10 of GAHT. Dividing people according to BO, a significant decline in Z-scores was found only in those who had undergone surgery at year 5 and at year 10. The literature reports scarce and incomplete data in this regard. Wiepjes et al. reported improved Z-scores at LS after 10 years of GAHT, but, in their study, people who had undergone gonadectomy were excluded and the FN or TH parameters were not reported (5). However, higher BMD in subjects with higher E2 levels was reported, and these data agree with our finding of higher E2 levels in the 5-year no-BO subgroup with stable BMD. Dobrolińska et al. found a decline in BMD and Z-score at the LS and TH sites in a small group of gonadectomized TGD AFAB individuals after 15 years of GAHT compared to those in the first 5 and 10 years. However, they do not report baseline data or longitudinal follow-ups for each subject, making definitive interpretation of that data difficult (21). The strength of our study is that, in the 5-year and 10-year subgroups, all individuals had baseline, 5- and 10-year bone parameters. Thus, a longitudinal analysis could be carried out. Miyajima et al. (9) found a more rapid decrease in LS BMD in operated transgender AFAB people compared to that in cisgender AFAB people undergoing spontaneous menopause. The decreased Z-scores that we found in our study may be due to the greater decline in Es levels after BO compared to those in transgender subjects who received GAHT and maintained their ovaries. The dose of exogenous T was not adjusted after surgery in these subjects, so this decrease may be due to the lack of the ovarian contribution to the production of Es. These results confirm previous studies reporting a decrease in circulating E2 (22) in TGD AFAB individuals after BO. The relationship between estradiol and BMD has been investigated, and it has been hypothesized that estradiol concentrations between 25 pg/mL and 40 pg/mL can effectively prevent bone loss (23, 24). However, whether this holds true even for the TGD AFAB population treated with T is even more uncertain. Our data in the 5-year BO subgroup compared to those in the 5-year no-BO subgroup seem also to support the presence of an E2 threshold for prevention of bone loss. However, the use of routine immunoassays that changed overtime and not of mass spectrometry methods to determine E2 levels (25) does not allow us to draw any conclusions on this issue. Another contributing factor may be the possible decreased adherence to GAHT at a time when, after hysterectomy and bilateral salpingo-oophorectomy, transgender AFAB people no longer fear the advent of menstrual bleeding. Suboptimal testosterone levels at the 10-year follow-up could support this hypothesis. Goh et al. (10) compared a group of transgender AFAB individuals who had undergone surgery and regularly took T to a similar group that had either discontinued or irregularly took T and found that the latter group showed worse BMD values. Unfortunately, in our study, we do not have data on the regularity of therapy intake.

With regard to the risk of fracture, in our study the main fractures reported by the subjects were associated with high-intensity traumas, whereas fragility fractures were negligible. All fractures occurred in individuals who had undergone BO. In 2019, Wiepjes et al. conducted a study on fracture risk, finding that a risk was comparable to reference cisgender women and reduced compared to reference cisgender men. They explained the latter finding by attributing it to a higher level of caution or lesser participation in sports associated with fracture risk compared to that in the reference cisgender men (26). The fractures of individuals enrolled in our study are proportionally higher in number compared to the study by Wiepjes et al.; however, in our study, the observation period for the occurrence of fractures ranges from the beginning of therapy to follow-up after 5 or 10 years of therapy. In the study of Wiepjes et al., the observation period covers only a 3-year window, which may explain the observation of a lower percentage of fracture events. However, the size of our sample and the rarity of fracture events make it impossible to obtain statistically significant data regarding the risk of fractures in our study.

In our study, all individuals who decided to have GAS underwent BO. Before 2015, in Italy, hysterectomy and BO were required in order to change one’s name on official documents. Therefore, most subjects chose to undergo complete genital surgery to obtain new documents. Now that the law has changed (27), transgender people have the option of avoiding surgery or to have their uterus removed with or without their ovaries. In counseling these subjects, therefore, it is very important to provide information on the risk-benefits of retaining the ovaries during GAS including the long-term effects on bone mass (28, 29). However, our data suggest a small decrease in Z-scores of all three bone sites in subjects who had undergone BO, but whether this decrease continues over time and leads to an increased fracture risk remains unknown.

The strength of this study is that it is one of the few studies with a long-term follow-up of up to 10 years, combining densitometric data with hormonal data and parameters of bone metabolism. It also provides implications in the context of surgical options that can be offered to transgender AFAB individuals, allowing for adequate counseling and tailored choice based on people’s needs.

We must acknowledge certain limitations within our study: the participant pool is relatively small, and the individuals enrolled are people attending a specialized center, making them likely to be more sensitive and attentive to medical follow-up. Moreover, the variations observed on densitometry parameters are significant, but their clinical impact in terms of increased fracture risk is unclear.

In conclusion, our study confirms that bone density in TGD AFAB individuals is comparable to that in their peers prior to GAHT and BO. However, these preliminary results also suggest that BO is associated with a reduced Z-score at LS, FN, and TH. These data, if confirmed in longer-term and larger-scale follow-ups, will be important in the surgical counseling of transgender AFAB people.

## Data Availability

The datasets will be available upon reasonable request to the authors. Requests to access these datasets should be directed to elenasanna.ca@gmail.com.

## References

[1] Hembree WC Cohen-Kettenis PT Gooren L Hannema SE Meyer WJ Murad MH. Endocrine treatment of gender-dysphoric/gender-incongruent persons: an endocrine society clinical practice guideline. J Clin Endocrinol Metab. (2017) 102:3869–903. doi: 10.1210/jc.2017-01658 28945902

[2] Coleman E Radix AE Bouman WP Brown GR de Vries ALC Deutsch MB. Standards of care for the health of transgender and gender diverse people, version 8. Int J Transgender Health. (2022) 23:S1–259. doi: 10.1080/26895269.2022.2100644 PMC9553112 36238954

[3] Van Caenegem E Wierckx K Taes Y Schreiner T Vandewalle S Toye K. Body composition, bone turnover, and bone mass in trans men during testosterone treatment: 1-year follow-up data from a prospective case-controlled study (ENIGI). Eur J Endocrinol. (2015) 172:163–71. doi: 10.1530/EJE-14-0586 25550352

[4] Wiepjes CM Vlot MC Klaver M Nota NM de Blok CJ de Jongh RT. Bone mineral density increases in trans persons after 1 year of hormonal treatment: A multicenter prospective observational study. J Bone Miner Res Off J Am Soc Bone Miner Res. (2017) 32:1252–60. doi: 10.1002/jbmr.3102 28370342

[5] Wiepjes CM de Jongh RT de Blok CJ Vlot MC Lips P Twisk JW. Bone safety during the first ten years of gender-affirming hormonal treatment in transwomen and transmen. J Bone Miner Res Off J Am Soc Bone Miner Res. (2019) 34:447–54. doi: 10.1002/jbmr.3612 PMC7816092 30537188

[6] Emmanuelle N-E Marie-Cécile V Florence Trémollieres Jean-François A Françoise L Coralie F. Critical role of estrogens on bone homeostasis in both male and female: from physiology to medical implications. Int J Mol Sci. (2021) 22:1568. doi: 10.3390/ijms22041568 33557249 PMC7913980

[7] Meriggiola MC Armillotta F Costantino A Altieri P Saad F Kalhorn T. Effects of testosterone undecanoate administered alone or in combination with letrozole or dutasteride in female to male transsexuals. J Sex Med. (2008) 5:2442–53. doi: 10.1111/j.1743-6109.2008.00909.x 18624972

[8] Giacomelli G Meriggiola MC. Bone health in transgender people: a narrative review. Ther Adv Endocrinol Metab. (2022) 13:20420188221099346. doi: 10.1177/20420188221099346 35651988 PMC9150228

[9] Miyajima T Kim YT Oda H. A study of changes in bone metabolism in cases of gender identity disorder. J Bone Miner Metab. (2012) 30:468–73. doi: 10.1007/s00774-011-0342-0 22222419

[10] Goh HH Ratnam SS. Effects of hormone deficiency, androgen therapy and calcium supplementation on bone mineral density in female transsexuals. Maturitas. (1997) 26:45–52. doi: 10.1016/S0378-5122(96)01073-0 9032746

[11] van Kesteren P Lips P Gooren LJ Asscheman H Megens J. Long-term follow-up of bone mineral density and bone metabolism in transsexuals treated with cross-sex hormones. Clin Endocrinol (Oxf). (1998) 48:347–54. doi: 10.1046/j.1365-2265.1998.00396.x 9578826

[12] World Health Organisation. WHO guidelines on physical activity and sedentary behaviour. Geneva: World Health Organization (2020). Available at: https://iris.who.int/bitstream/handle/10665/336656/9789240015128-eng.pdf?sequence=1. Licence: CC BY-NC-SA 3.0 IGO.

[13] Meriggiola MC Costantino A Cerpolini S Bremner WJ Huebler D Morselli-Labate AM. Testosterone undecanoate maintains spermatogenic suppression induced by cyproterone acetate plus testosterone undecanoate in normal men. J Clin Endocrinol Metab. (2003) 88:5818–26. doi: 10.1210/jc.2003-030574 14671175

[14] Pelusi C Costantino A Cerpolini S Pelusi G Meriggiola MC Pasquali R. A placebo-controlled, randomized clinical trial using testosterone undecanoate with injectable norethisterone enanthate: effect on anthropometric, metabolic and biochemical parameters. Int J Androl. (2011) 34:548–55. doi: 10.1111/j.1365-2605.2010.01122.x 21087288

[15] Gava G Cerpolini S Martelli V Battista G Seracchioli R Meriggiola MC. Cyproterone acetate vs leuprolide acetate in combination with transdermal oestradiol in transwomen: a comparison of safety and effectiveness. Clin Endocrinol (Oxf). (2016) 85:239–46. doi: 10.1111/cen.13050 26932202

[16] Gava G Mancini I Orsili I Bertelloni S Alvisi S Seracchioli R. Bone mineral density, body composition and metabolic profiles in adult women with complete androgen insensitivity syndrome and removed gonads using oral or transdermal estrogens. Eur J Endocrinol. (2019) 181:711–8. doi: 10.1530/EJE-19-0383 31491747

[17] Gava G Mancini I Alvisi S Seracchioli R Meriggiola MC. A comparison of 5-year administration of cyproterone acetate or leuprolide acetate in combination with estradiol in transwomen. Eur J Endocrinol. (2020) 183:561–9. doi: 10.1530/EJE-20-0370 33055297

[18] Gava G Armillotta F Pillastrini P Giagio S Alvisi S Mancini I. A randomized double-blind placebo-controlled pilot trial on the effects of testosterone undecanoate plus dutasteride or placebo on muscle strength, body composition, and metabolic profile in transmen. J Sex Med. (2021) 18:646–55. doi: 10.1016/j.jsxm.2020.12.015 33531255

[19] Guney E Kisakol G Ozgen G Yilmaz C Yilmaz R Kabalak T. Effect of weight loss on bone metabolism: comparison of vertical banded gastroplasty and medical intervention. Obes Surg. (2003) 13:383–8. doi: 10.1381/096089203765887705 12841898

[20] Strope MA Nigh P Carter MI Lin N Jiang J Hinton PS. Physical activity-associated bone loading during adolescence and young adulthood is positively associated with adult bone mineral density in men. Am J Mens Health. (2015) 9:442–50. doi: 10.1177/1557988314549749 25237041

[21] Dobrolińska M van der Tuuk K Vink P van den Berg M Schuringa A Monroy-Gonzalez AG. Bone mineral density in transgender individuals after gonadectomy and long-term gender-affirming hormonal treatment. J Sex Med. (2019) 16:1469–77. doi: 10.1016/j.jsxm.2019.06.006 31326306

[22] Kumar S Bertin E O’Dwyer C Khorrami A Wassersug R Mukherjee S. Serum estradiol levels decrease after oophorectomy in transmasculine individuals on testosterone therapy. Asian J Androl. (2023) 25:309–13. doi: 10.4103/aja202262 PMC10226504 36124534

[23] Barbieri CE. Editorial comment. What factors influence estradiol levels in women receiving postmenopausal hormone therapy?. NEJM, Journal Watch. Summary and comment. Women's health informing practice. Vol. 204. (2020). pp. 712–3.

[24] Wang N Zhang Y Huang C. Association between Estradiol and Bone Mineral Density in Adults Aged 40-60 years (2022). Available online at: https://www.researchsquare.com/article/rs-1355824/v1 (Accessed March 28, 2022).

[25] Handelsman DJ Wartofsky L. Requirement for mass spectrometry sex steroid assays in the Journal of Clinical Endocrinology and Metabolism. J Clin Endocrinol Metab. (2013) 98:3971–3. doi: 10.1210/jc.2013-3375 24098015

[26] Wiepjes CM de Blok CJ Staphorsius AS Nota NM Vlot MC de Jongh RT. Fracture risk in trans women and trans men using long-term gender-affirming hormonal treatment: A nationwide cohort study. J Bone Miner Res Off J Am Soc Bone Miner Res. (2020) 35:64–70. doi: 10.1002/jbmr.3862 PMC7003754 31487065

[27] Supreme court of cassation. Civil law, section 1, decree 20/07/2015 . Available online at: https://www.gazzettaufficiale.it/atto/corte_costituzionale/caricaArticolo?art.progressivo=0&art.idArticolo=3&art.versione=1&art.codiceRedazionale=T-150221&art.dataPubblicazioneGazzetta=2015-11-11&art.idSottoArticolo=0 (Accessed March 28, 2022).

[28] Kumar S Mukherjee S O’Dwyer C Wassersug R Bertin E Mehra N. Health outcomes associated with having an oophorectomy versus retaining one’s ovaries for transmasculine and gender diverse individuals treated with testosterone therapy: A systematic review. Sex Med Rev. (2022) 10:636–47. doi: 10.1016/j.sxmr.2022.03.003 35831234

[29] Grimstad FW Fraiman E Garborcauskas G Ferrando CA. Retrospective review of changes in testosterone dosing and physiologic parameters in transgender and gender-diverse individuals following hysterectomy with and without oophorectomy. J Sex Med. (2023) 20:690–8. doi: 10.1093/jsxmed/qdad031 36987750

