# Supplementary material for: Bone health in transgender assigned female at birth people: effects of gender-affirming hormone therapy and gonadectomy
Source: Front Endocrinol (Lausanne). 2024 Sep 26;15:1416121. doi: 10.3389/fendo.2024.1416121 (PMC11464845; doi:10.3389/fendo.2024.1416121)

Supplementary Materials

Figure 1: linear correlation between LS-BMD and BMI (A), LS-Z score and BMI (B), TH-BMD and BMI (C), TH-Z score and BMI (D), FN-BMD and BMI (E), FN-Z score and BMI (F).

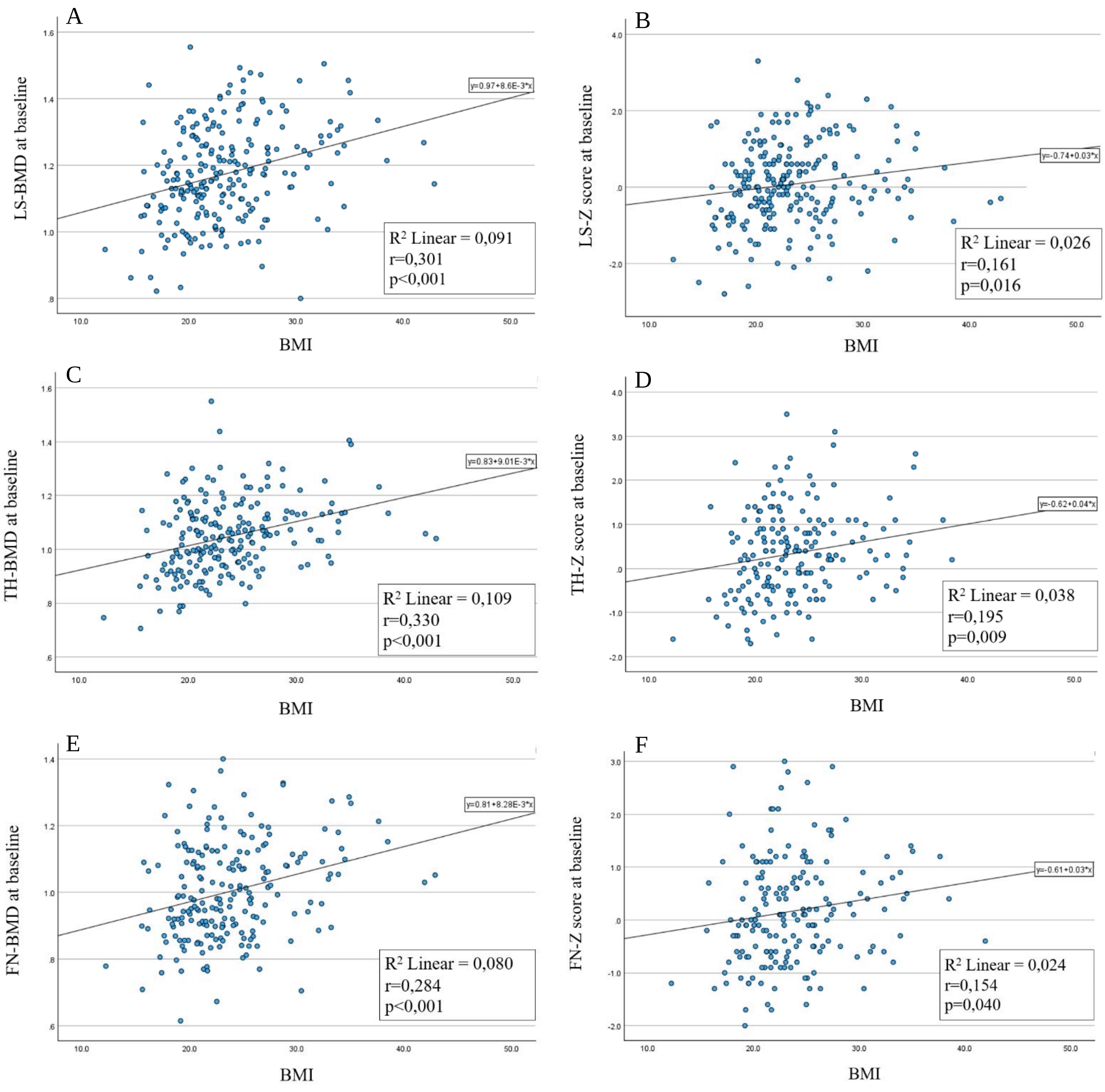

Supplement: Supplementary file 1 [file DataSheet1.pdf]
